# Global shocks, cascading disruptions, and (re-)connections: viewing the COVID-19 pandemic as concurrent natural experiments to understand land system dynamics

**DOI:** 10.1007/s10980-023-01604-2

**Published:** 2023-03-02

**Authors:** María Piquer-Rodríguez, Cecilie Friis, R. Ntsiva N. Andriatsitohaina, Sébastien Boillat, Paula Roig-Boixeda, Chiara Cortinovis, Davide Geneletti, Maria-Jose Ibarrola-Rivas, Lisa C. Kelley, Jorge C. Llopis, Elizabeth A. Mack, Ana Sofía Nanni, Julie G. Zaehringer, Geoffrey M. Henebry

**Affiliations:** 1 Institute of Geographical Sciences, Freie Universität Berlin, Berlin, Germany; 2 Department of Geosciences and Natural Resource Management, University of Copenhagen, Copenhagen, Denmark; 3 Department of Forestry and Environment, School of Agronomy Sciences, University of Antananarivo, Antananarivo, Madagascar; 4 Institute of Geography, University of Bern, Bern, Switzerland; 5 School of Agricultural, Forest and Food Sciences HAFL, Bern University of Applied Sciences, Zollikofen, Switzerland; 6 Institute of Biology, University of Neuchâtel, Neuchâtel, Switzerland; 7 Frankfurt Zoological Society, Bernhard-Grzimek-Allee 1, 60316 Frankfurt Am Main, Germany; 8 Institute of Environmental Science and Technology, Universitat Autònoma de Barcelona, 08193 Bellaterra, Spain; 9 Department of Geography, Humboldt-Universität zu Berlin, Berlin, Germany; 10 Department of Civil, Environmental and Mechanical Engineering, University of Trento, Trento, Italy; 11 Institute of Geography, Universidad Nacional Autónoma de México (UNAM), Mexico City, Mexico; 12 Department of Geography and Environmental Sciences, University of Colorado, Denver, Denver, CO USA; 13 School of Natural Sciences, Bangor University, Bangor, UK; 14 Centre for Development and Environment, University of Bern, Bern, Switzerland; 15 Department of Geography, Environment, and Spatial Sciences, Michigan State University, East Lansing, MI USA; 16 Instituto de Ecología Regional and Facultad de Ciencias Naturales e IML, Universidad Nacional de Tucumán, Tucumán, Argentina; 17 Wyss Academy for Nature at the University of Bern, Bern, Switzerland; 18 Institute of Geography, University of Bern, Hallerstrasse 12, 3012 Bern, Switzerland; 19 Department of Geography, Environment, and Spatial Sciences and Center for Global Change and Earth Observations, Michigan State University, East Lansing, MI USA

**Keywords:** Socio-ecological land systems, Resilience, Telecoupling, Mobility, Governance, Conservation

## Abstract

**Context:**

For nearly three years, the COVID-19 pandemic has disrupted human well-being and livelihoods, communities, and economies in myriad ways with consequences for social-ecological systems across the planet. The pandemic represents a global shock in multiple dimensions that has already, and is likely to continue to have, far-reaching effects on land systems and on those depending on them for their livelihoods.

**Objectives:**

We focus on the observed effects of the pandemic on landscapes and people composing diverse land systems across the globe.

**Methods:**

We highlight the interrelated impacts of the pandemic shock on the economic, health, and mobility dimensions of land systems using six vignettes from different land systems on four continents, analyzed through the lens of socio-ecological resilience and the telecoupling framework. We present preliminary comparative insights gathered through interviews, surveys, key informants, and authors’ observations and propose new research avenues for land system scientists.

**Results:**

The pandemic’s effects have been unevenly distributed, context-specific, and dependent on the multiple connections that link land systems across the globe.

**Conclusions:**

We argue that the pandemic presents concurrent “natural experiments” that can advance our understanding of the intricate ways in which global shocks produce direct, indirect, and spillover effects on local and regional landscapes and land systems. These propagating shock effects disrupt existing connections, forge new connections, and re-establish former connections between peoples, landscapes, and land systems.

**Supplementary Information:**

The online version contains supplementary material available at 10.1007/s10980-023-01604-2.

## Introduction

The COVID-19 pandemic has spread across the globe over the course of the past two years, infecting well over half a billion people and resulting in the death of at least 6.7 million as of January 2023 (JHU 2023). Beyond its direct effects on human health, the manifold disturbances to economies, institutions, societies, and established political systems and policy processes have made the COVID-19 pandemic one of the most disruptive global shocks since the World Wars (Maital and Barzani 2020). It is, perhaps, the first truly global shock in the early twenty-first century. Countless national, regional, and local implementations of border closures, lockdowns, social distancing, and other containment policies (Teachout and Zipfel 2020; Hale et al. 2021), ongoing production and supply chain shocks (Padhan and Prabheesh 2021), and altered consumption preferences and patterns (Kwon et al. 2022) have destabilized national and regional economies, triggering new manifestations of inequality, political instability, and conflict (Bloem and Salemi 2021; Ahmed et al. 2022). These burdens have fallen on families and individuals in gendered, generational, and racialized ways (Myers et al. 2020; Agarwal 2021), in the form of lost jobs and income streams, return migration from cities to villages (Marschke et al. 2021; Suhardiman et al. 2021), and strained access to food, water, and health care (Mishra and Rampal 2020; Collins et al. 2021), among others.

Despite growing understanding of the pandemic’s multi-faceted implications, the links to land system dynamics have remained relatively underexplored. So far, emerging research on the COVID-19 pandemic and land systems has focused mainly on the relation between land-use change and the potential for new pathogen emergence (Azevedo et al. 2020; Gibb et al. 2020). Research on how the pandemic feeds back into land systems through the disruption of existing conditions or the generation of new connections remains scarce (Boillat and Zaehringer 2020; Brancalion et al. 2020; Nolte et al. 2022). Addressing this knowledge gap is key for three reasons. First, land use and land users are related in complex and contingent ways to global production, processing, and delivery systems, labor networks, and markets and, critically, to the landscapes and ecosystems that host the land uses. Thus, we expect that land systems will be affected by disruptions or reconfigurations of these systems, networks, and markets. Second, many families, communities, and even nations rely on labor migration to support strained household economics through remittances to lower and middle income countries) and to sustain industrialized forestry, fuel, fiber, and food operations through provision of labor (Radel et al. 2019). The pandemic-related disruption of the movement of both people and products has in some cases raised the potential for conflicts and competition for land between, for example, subsistence agriculture and production of export commodities. Third, it is widely acknowledged that the risks and burdens of disease are unevenly distributed and absorbed, both socially and spatially (Bathina et al. 2021). Thus, place-based studies and comparative work on pandemic-land relations are needed to characterize trajectories of change and (re-)connectivity and the commonalities and differences in such trajectories across locations (e.g., Sawalhah et al. 2021; Chin et al. 2020).

This perspective article emerged from a series of webinars and online discussions held under the aegis of the Global Land Programme (https://glp.earth) during the latter half of 2021. GLP members shared their insights and experiences on two topics: (i) the effects (detected or expected) of the COVID-19 pandemic on the land systems they study; and (ii) the frameworks, approaches, and tools that could be used to detect and evaluate such changes. The subsequent discussions offered a kaleidoscope of insights, and experiences gleaned from a variety of land systems and the impacts of the pandemic. We formalized these discussions through a collaborative exercise of brainstorming sessions where we shared our perceptions and lessons from each other's land systems. This article summarizes the outcomes of those sessions, with the aim to stimulate and encourage fellow land systems scientists to take advantage of this highly unusual global event to advance understanding of land system interactions, responses to systemic shocks, subsequent developmental trajectories, and opportunities for adaptive management.

**Fig. 1:**
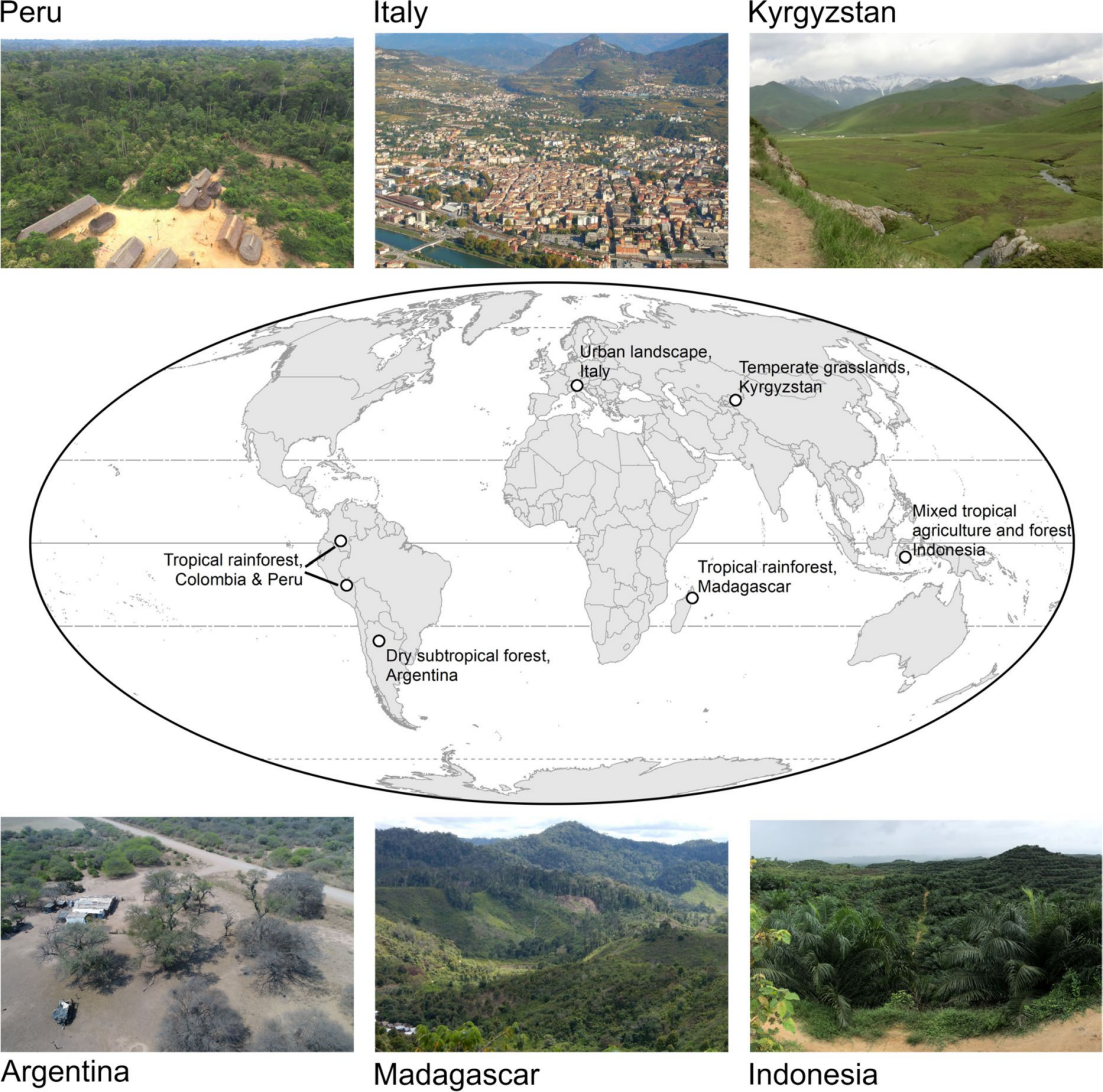
Location of the six vignettes illustrating COVID-19 impacts on land systems and a photo of a representative landscape. Clockwise from top left: Matsiguenka village in the Peruvian Amazon rainforest near Manu National Park (photo credit: D. Rosengren); urban landscape of Trento, Italy (photo credit: D. Geneletti), temperate montane agropastoralist landscape in Naryn, Kyrgyzstan (photo credit: G. Henebry); mixed agribusiness and smallholder landscape in Besulutu, Sulawesi, Indonesia (photo credit: L. Kelley); Betsimisaraka village by the northeastern Madagascar rainforest near Makira Natural Park (photo credit: J. Llopis); agribusiness landscape in the Chaco Dry forests of northern Argentina (photo credit: B. Gobbi)

We synthesized key insights from the pandemic shocks to geographically diverse land systems using six vignettes from land systems in the Amazon Basin (Colombia and Peru), Argentina, Madagascar, Indonesia, Kyrgyzstan, and Italy (Fig. 1). The six vignettes were first presented during the GLP webinars. The vignettes span four continents, and include both tropical and temperate ecosystems, rural and urban areas, and diverse spatial extents (see SI for more details). Although the selection of the vignettes was opportunistic rather than systematic, and the glimpses into these diverse land systems was necessarily limited, the co-production of this piece enabled us to share and engage in an extended interdisciplinary discussion on the impacts of a major global shock on different land systems. Each vignette draws on expert knowledge and includes insights from direct observations and fieldwork by the authors in the narrow window for local and international travel possible during summers of 2021 and 2022, or from reports and conversations with research partners, key informants, local stakeholders, media reports and other online resources explored in the last three years.

We build on three conceptual frameworks to describe the responses and interactions triggered by the COVID-19 pandemic in the six vignettes. First, the effects of *shocks* on land-use change and land-use decisions are well documented (Ramankutty and Coomes 2016). They include, for example, effects of institutional shocks (de Beurs and Henebry 2004; Prishchepov et al. 2012), warfare and armed conflicts (de Beurs and Henebry 2008; Baumann and Kuemmerle 2016) on land abandonment, shifts in agricultural land use (Ioffe et al. 2012; Schierhorn et al. 2013), introduction of new regulations and subsidies (Bartolini and Viaggi 2013; Quiroga et al. 2017), and economic shocks leading to deforestation and/or widespread shifts in land control and access (Pagiola 2000; Robertson and Pinstrup-Andersen 2010; Spalding 2017; Fairbairn 2020).

Second, the theory of social-ecological systems resilience (Walker et al. 2004; Folke 2006) presents valuable heuristic tools to organize the complexity of land systems that evolve and respond to shocks. While much attention has been given to shocks and crises as destructive forces, research on systems resilience also emphasizes how crises can offer windows of opportunities for innovations and new forms of coordination that may lead to strengthening of the adaptive capacity to stress and future shocks (Holling 2001; Wu 2006; Brown 2015; Cumming and Peterson 2017).

Third, the telecoupling framework captures the social-ecological interactions over distances that characterize land systems under globalization and global environmental change (Liu et al. 2013; Friis and Nielsen 2019). From this perspective, the pandemic is a global external force influencing land systems by generating new connections and disrupting or transforming existing ones. The telecoupling framework facilitates identification of direct effects on land systems associated with the pandemic (i.e., a land system change that has a direct cause in the pandemic), indirect impacts on receiving systems (i.e., impacts on land-use in one place which are caused by land-use changes in another place), and potential spillover and feedback effects (i.e., land-use changes in one place have impacts on land use and other land systems in another place, which can, in turn, impact back into the original land system) (Liu et al. 2018; Meyfroidt et al. 2020).

## Pandemic effects and feedbacks in six land systems

### Amazon Basin: Peru and Colombia

This vignette focuses on two protected landscapes in the Amazon basin (an extensive protected area network in southeastern Peru and a protected landscape in southern Colombia) and is based on reports by key informants working for the conservation of these areas. (See S1 for additional information about this study area and the others.) Countries in the Amazon basin, such as Colombia and Peru, were severely hit by the COVID-19 pandemic, and for some time recorded among the highest numbers of cases and deaths worldwide (Mathieu et al. 2020). There was fear that the disease could be devastating for the indigenous people (Moeller and Pedersen 2020). In response to that threat and as an example of solidarity emerging during the pandemic, both governmental and non-governmental conservation actors went beyond their designated roles to provide preventive and curative health support to indigenous communities (FZS 2020, 2021). The countries also implemented stringent and long-lasting restrictions to contain the pandemic, including lockdowns, international and national mobility restrictions and school closures (Mathieu et al. 2020). Biodiversity conservation efforts in two protected area networks in the Amazon basin (southeastern Peru and southern Colombia) were disrupted by national restrictions on mobility aimed to limit the spread of COVID-19, thereby disrupting local and regional economies and affecting conservation workers and members of indigenous communities. The immediate economic burdens of the pandemic shifted budgetary priorities, reducing domestic conservation budgets. Some international conservation NGOs maintained or even increased their support to alleviate the gaps left by reduced government funding. Nevertheless, domestic budget cuts together with the disease and mobility restrictions affecting conservation workers hindered monitoring and law enforcement efforts. This situation, combined with increased demand and value of commodities such as gold, is believed to have aggravated a pre-existing governance vacuum that was leveraged by illegal resource users and criminal groups and led to increased criminality, ecological degradation, land/forest conversion. There are reports and evidence of increases in illegal appropriation of natural resources, e.g., hunting, expansion of agricultural and cattle grazing areas, logging, drug cultivation and trafficking, and alluvial gold mining, all leading to deforestation (Céspedes et al. 2022; Roig-Boixeda, unpublished data). Overall, the pandemic appears to be a multiplier of existing threats and pressures, rather than a generator of new ones. A major concern among conservation actors has been that rolling back the increased expansion of criminal actors and illegal activities will be difficult post-pandemic. It is also feared that disrupted local economies, including those dependent on international tourism in the protected areas, could be contributing to this trend or do so in the near future. Mobility restrictions at the local, national, and international scales constituted another pandemic-triggered stressor and affected the two protected area networks both negatively and positively. For instance, movement restrictions led to reduced on-the- ground presence of conservation actors, thereby contributing to the goverance vacuum. In response to these restrictions, many activities and meetings were moved online, requiring access to reliable internet. Whereas this “digitalization” prevented a complete disruption of conservation processes and communications, it may have suppressed participation by members of indigenous communities with limited digital capacity. On the other hand, indigenous people, however, were not affected by mobility restrictions and continued to engage in conservation activities within the protected areas. This situation highlights their key role and positionality as conservation allies. Finally, a positive consequence of lockdowns was reported for some highly-visited protected areas: increased sighting of fauna, including unusual species, likely due to the reduced human presence and movement (Roig-Boixeda, unpublished data).

### Dry Chaco, Argentina

The Argentine Dry Chaco includes most of the South American Gran Chaco and harbors the largest subtropical dry forests in the world. It is also a deforestation hotspot where agriculture and pastureland, mainly for soybean production and cattle ranching, have expanded rapidly in the last decades, affecting natural resources and the livelihoods that rely on them (Baumann et al. 2017; Levers et al. 2021). At the same time, poverty and inequality are very high in the Dry Chaco, particularly in more rural areas (Torrella and Adámoli 2005), for which the pandemic deepened pre-existing socio-economic issues, and particularly influenced already vulnerable rural communities (Rubio and Córdoba 2020). As in many places, two major direct effects of COVID-19 experienced in this region were economic disruptions and mobility restrictions (Figure S1).

The pandemic’s economic burdens coupled with a pre-existing national recession affected government budgets and their priorities. Similar to the Amazon Basin, these budget cuts together with mobility restrictions hindered governmental control and enforcement for conservation in the Dry Chaco. Mobility restrictions at local, national, and international levels were not equally applied across all sectors, leading to heterogeneous impacts on societal actors. For instance, the need to continue the production of agricultural commodities led to the Argentine government rapidly easing mobility restrictions for agriculture. This early movement of agricultural workers may have facilitated the spread of the virus into rural areas with precarious health systems, such as remote forests or villages inhabited by local communities. Some communities self-organized to block incoming workers to limit disease spread.

Lockdowns had at least two negative effects on conservation. First, the reduced presence of government authorities jeopardized implementation and enforcement of existing regulations, and may have facilitated unsanctioned appropriation of natural resources (*e.g.*, hunting, logging, unauthorized expansion of agriculture and cattle grazing). A recent study documenting agricultural frontier expansion in the South American Chaco shows a surge in deforestation after 2019 (Baumann et al. 2022). This implies increasing forest conversion during the lockdown period, mirroring the situation of other deforestation frontiers globally (Fairbairn 2020; Céspedes et al. 2022). Second, mobility restrictions limited access to both researchers and NGOs conducting conservation activities, thereby disrupting ongoing conservation projects and initiatives. Similar to other parts of the world, restrictions led to the need to improve remote communication. Some farms and rural areas of the Dry Chaco benefited from these improvements, an example of which is an increase of remote monitoring of agricultural activities and facilitated interactions between farm owners—who usually live elsewhere—and their employees (Nanni, *personal observation*). However, inequalities in access to improved digital communications tools further marginalized residents with limited access to the internet. In many small villages of the Chaco, the implementation of remote learning precluded many students from continuing their studies because of lack of connectivity (Rubio and Córdoba 2020), as reported throughout rural territories of Argentina (Alcoba et al. 2021).

### Maroantsetra, Madagascar

When the three first cases of COVID-19 were confirmed in Madagascar in March 2020, the government closed the national borders in an attempt to halt the spread of the disease on the island. This immediately halted international tourism, a critical sector of the country’s economy, including financial support for protected area management (Goodman et al. 2018). The government also restricted movement between regions to prevent the spread of COVID-19 to rural areas. Despite these steps, an unknown but potentially substantial number of urban workers left the capital, returning to family in the countryside (The Citizen 2020).

These measures were also felt in the remote north-eastern Maroantsetra District, home to some of the largest protected areas in Madagascar, featuring biodiversity-rich, moist forests. Masoala National Park, for instance, registered fewer than 70 international visitors in 2020—2% of the expected number—forcing guides and people with tourism-dependent livelihoods to sell assets in order to cope with the income loss. Moreover, the region is one of the world’s major vanilla producing areas, a critical cash crop for rural populations (Osterhoudt 2017), whose international market price has plummeted following record prices between 2013 and 2018 (Terazono 2017). Anecdotal evidence suggests that the combined effect of these factors might have forced rural populations to rely more on subsistence agriculture—such as shifting cultivation for rice production—or to move into artisanal quartz crystal mining, which has undergone several boom and bust cycles in the region since the early 2000s (Holmes 2007). Implications of these changing conditions for local livelihoods and the landscapes that host them remain to be investigated. However, it is likely that pressure on the region’s forests has been increasing, given that both shifting cultivation and artisanal mining are often expanded into remaining forests (Cook and Healy 2012; Zaehringer et al. 2015), and this has been central in strategies deployed by local populations in the face of past shocks (Llopis et al. 2019). Evidence from protected areas across the country indeed suggests that pressure on the island’s forest may have increased during the peak of the pandemic, when effective management of protected areas was more difficult to deliver (Eklund et al. 2022). To address the most acute effects of the pandemic and with an eye to future crises, conservation scientists and practitioners in Madagascar suggest reinforcing support for resilient local livelihoods and investment in local capacity building as a way forward (Razanatsoa et al. 2021).

### Besulutu, Indonesia

Besulutu, a small sub-district (96 km^2^) located roughly an hour’s drive from Sulawesi, Indonesia’s eastern coast, holds considerable biocultural significance to the Indigenous Tolaki people. Once encompassing extensive floodplains, agro-forests, rice fields, montane forests, and marshes, the area has experienced substantial landscape homogenization since the 1980s. Industrial nickel mining and corporate oil palm concessionaires hold long-term use rights to ~ 70% of all land in Besulutu as of 2020, and Indonesia’s Ministry of Forestry controls another 20% as part of its forest reserves (Dean 2021). Households manage 0.5–1.5 ha of land for seasonal vegetable crops, daily uses, and commodity production, and most depend on work outside the village in some form and/or on work at local oil palm and mining concessions.

To understand pandemic-land relations in Besulutu, informal and in-depth interviews were conducted in October 2021 with farmers, workers, villagers, and government officials (Kelley, unpublished data). Results show that mobility restrictions were perceived to be a larger source of COVID-19-linked harm than the immediate effects of the disease. Three waves of such restrictions during periods of high COVID-19 incidence since March 2020 interrupted access to urban and peri-urban labor markets and trade networks for months at a time, necessitating the closure of many female-operated food stalls and businesses within villages and along nearby roads.

Returning labor migrants temporarily resolved pre-existing agricultural labor shortages in villages and facilitated plantings of patchouli, maize, and oil palm in remaining fields using government COVID-19 relief funds to purchase seeds and other inputs. COVID-19 led to company and government policies to prioritize recruitment of local workers, which, depending on village location, unevenly informed both work openings and layoffs. Interviews also indicate the need to attend to questions of social differentiation and concurrent land-livelihood ‘shocks’ in future work. For instance, heavy rains and floods since 2018 routinely cut off access to work on plantations and mines, negatively affect new commodity crop plantings, and shape gendered and generational challenges in accessing clean food and water during times of stress (Kelley et al. 2022).

### Naryn, Kyrgyzstan

In Kyrgyzstan, a small and highly mountainous country in Central Asia, most of the 6.3 million inhabitants live in rural areas. Montane agropastoralism is the basis of the rural economy, featuring seasonal movements of livestock to more distant, higher elevation pastures coupled with cultivation of forage for winter feed. Kyrgyzstan is heavily dependent on remittances sent home by labor migrants, who come mostly from rural areas. International remittances averaged 29% of Kyrgyzstan’s GDP between 2010 and 2017 (Mack et al. 2021).

In rural Naryn oblast during July 2021 and in rural Osh oblast in July 2022, we gathered information about experiences with COVID-19 and its effects through formal surveys, in-depth interviews, and informal anecdotes with villagers, herders, and government officials (Mack, *unpublished data*). A first wave of COVID-19 swept through the province during summer 2020 with the source of transmission being villagers who had traveled to the capital city on business (*e.g.*, to sell livestock products or purchase supplies). Health infrastructure in rural Kyrgyzstan is limited. Many got sick with COVID-19: some elderly died from the disease; most of the infected recovered without severe consequences. However, the health data are uncertain and incomplete. By all accounts, the principal impacts of the pandemic and associated lockdown were economic: lost wages, lost jobs, lost revenue, limited supplies of food, medicine, agricultural inputs, limited opportunity to sell products, and higher prices for production inputs and consumptive goods. Although we witnessed a reduction in tourism in Naryn in 2021 relative to earlier years that arose, in part, due to international perceptions about available health care (Saidmamatov et al. 2021), a partial recovery was evident in Osh during 2022. We did not witness nor heard from herders that the pandemic directly affected grazing duration or intensity. This response was not surprising given the high interannual variation in the weather at these elevations.

Some respondents indicated the pandemic paused migration activity, and the cessation of remittance flows during the pandemic had affected them financially. Contrary to expectation, there was not a significant reduction in remittances to Kyrgyzstan at the national scale between 2019 and 2020, but instead a modest 0.5% increase and a substantial increase of more than 15% increase between 2020 and 2021 (World Bank-KNOMAD 2022). Only the 2019–2020 increase was weak compared to the 13% average annual rate of increase in remittances to Kyrgyzstan 2010–2017. More work remains to understand regional variation in remittance dynamics. The picture emerging at the national level may not reflect the lived experience in vulnerable households who are more likely to send labor migrants as a livelihood strategy (Sagynbekova 2017).

### Trento, Italy

Trento is a city of 120,000 inhabitants in the Eastern Alps. The urban landscape is characterized by a main settlement with a relatively dense historical center, located on flat terrain in the valley floor of the Adige River, and by small villages scattered in the surrounding hillsides. Trento illustrates how local mobility restrictions, introduced to guarantee physical distancing, affected the opportunities to visit green spaces. Demand for nature-based recreation peaked during the pandemic (Venter et al. 2020; Derks et al. 2020). However, in many cities, access to urban parks was banned or restricted to those living in the immediate vicinity, thereby exacerbating an already difficult situation. Physical and mental health benefits of contact with nature are well documented (WHO 2017), and access to public green spaces is especially important for vulnerable groups (Gascon et al. 2015; Lee and Lee 2019).

In Trento, we applied GIS modeling to visualize and quantify the direct and indirect effects of different restriction policies (Geneletti et al. 2022). Our results revealed that limiting the distance from home that people were allowed to travel and forcing them to visit only the closest green space can result in a different distribution of users compared to pre-pandemic times. Large parks, which offer more natural settings and a wider range of activities, became less accessible and underutilized. In contrast, centrally located small parks—especially playgrounds—are likely to receive more users, and become overcrowded during peak hours.

A perception of reduced safety due to park overcrowding and an increased demand for recreational space pressured local governments to enhance the availability of green spaces. In Trento, we tested the potential benefits of transforming school yards into “emergency parks”. School yards offer the advantage of being publicly owned and evenly distributed in the different parts of the city. Our simulation demonstrated that adding school yards to the available green areas can increase the number of people with access to an uncrowded space, even though it requires higher maintenance and surveillance costs. In contrast, reducing overcrowding by limiting travel distance would decrease potential park users and increase existing inequalities, especially in densely-populated neighborhoods.

## Synthesis and future research

**Fig. 2:**
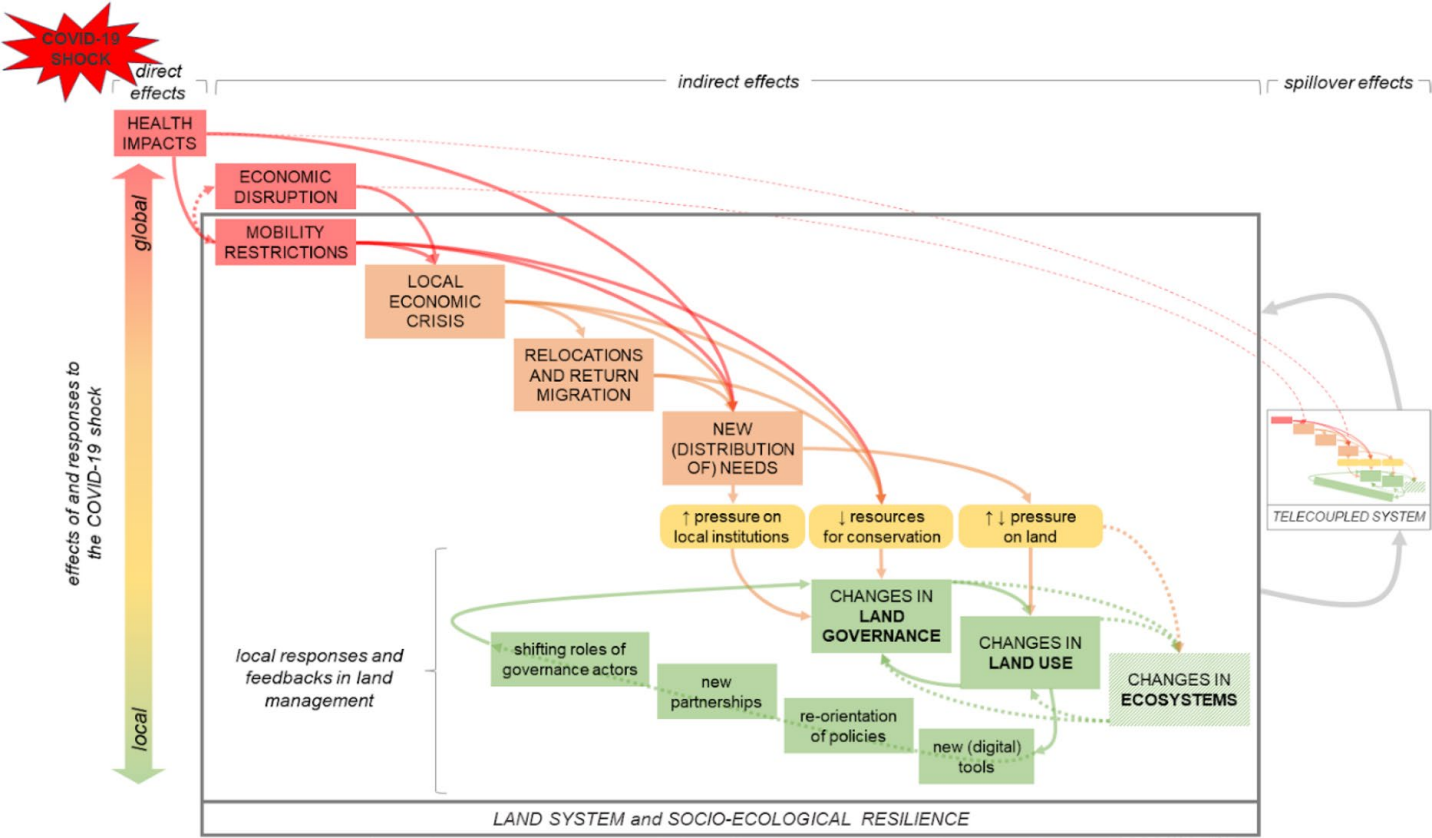
Illustrates the propagation of the effects from the global shock of the pandemic to changes in local land use and land governance, including only generalized effects and responses common to some vignettes. The cascade of disruptions highlights the complexity of linkages between multiple processes at different scales, pointing to the need for a multidimensional, multiscale approach to analyze them

The COVID-19 pandemic has had devastating human and social consequences for many people during the past three years. At the same time, the extent and unprecedented nature of the pandemic has generated concurrent “natural experiments of opportunity” (Craig et al. 2017) that can help us understand how social-ecological system connections change, how resilient they are to global shocks, and the relations between immediate and long-term effects of such shocks. The shock of the COVID-19 pandemic triggered interlinked cascading disruptions in land systems across different scales (Fig. 2). Besides the direct effects on human health, the pandemic’s principal indirect effects included mobility restrictions and economic disruptions leading to multiple effects across different sectors. These cross-cutting effects, sometimes aggravated by pre-existing social-ecological threats, triggered local economic crises, disrupting income and livelihoods, as was seen in loss of tourism revenues in Madagascar (2.3), displaced rural–urban migrants in Indonesia (2.4), rural producers restricted from access to urban markets in Kyrgyzstan (2.5), and population movements despite restrictions reported in Madagascar (2.3), Indonesia (2.4), and globally (FAO 2021). However, there were also some positive outcomes emerging from these disruptions, such as a (temporary) increase in fauna sightings in natural areas of the Amazon (2.1, S1.1), improved digital tools and remote communicaiton (albeit with unequal access) in Chaco (2.2, S1.2), and potential longer-term benefits of opening school playgrounds to the public in Trento (2.6, S1.6).

The combined effects of return migration, local economic crises, mobility restrictions, and health consequences have generated new distributions of land-use needs that induced altered land-use pressures (Fig. 2): fewer tourists in the Amazon and Madagascar led to reduced income and could have indirectly led to increased pressure on forest and land; limiting mobility led to reduced use of peripheral urban green spaces in Trento; returning labor migrants facilitated new commodity crop plantings in Indonesia; and a return to subsistence agriculture and artisanal mining in Madagascar. These changes have, in turn, pressured local institutions to adjust land governance, and led to responses at both local and national levels. In Indonesia, return migration and an increased agrarian labor supply led local leaders to invest COVID-19 relief funds in smallholder agricultural production. In Trento, changing access rules and management of public green areas emerged as a potential emergency solution to maintain mental health. However, in other areas, such as the Amazon and the Argentine Dry Chaco, decreased conservation budgets forced changes in practical conservation management, reducing enforcement and control activities that led to increased illegal appropriation of natural resources. Yet, in the Amazon, the restructuring of networks of actors and the use of new digital tools has offered new opportunities for conservation and forest management. Immediate changes in conservation enforcement in the wake of the pandemic may thus have longer-term ramifications for landscape change, ecosystem services, and land governance that merit further study. In addition to the direct and indirect effects of the pandemic, some of the observed phenomena—such as the early lifting of mobility restrictions in some commodity production regions in the Argentine Dry Chaco or the travel restrictions on areas dependent on tourism such as in Madagascar—point to spillover effects within and beyond the land systems studied (Fig. 2). These potential spillovers might include faster disease spread in agriculture workers to remote areas with weak health systems as in Argentina or increased deforestation from the expansion of subsistence agriculture due to a lack of tourism employment as in Madagascar. Further research is needed to identify, map, and understand these interactions.

Based on our six vignettes and the direct, indirect, and potential spillover effects of the pandemic on the land systems they represent, we see a number of important avenues for future research that can advance the intertwined disciplines of land system science, landscape ecology, and landscape sustainability science.

First, we need more research emphasis on the longer term effects of the direct and indirect pandemic impacts on land systems. What will, for example, be the longer term effects of reduced conservation budgets on the prevalence of deforestation and land grabbing, as seen in the Amazon and the Dry Chaco? How will the loss of wages and jobs affect local economies in the future and what will be the implications for local land uses and management practices? Will shifts in livelihood strategies consolidate after the pandemic with continuing increased pressure on forest resources, as seen with the subsistence farmers and small-scale miners in Madagascar? What will be the longer-term impacts of changing migration, mobility, and remittance trends? Will labor migrants return to cities, change their destinations, or remain in rural areas and, for example, continue to resolve agribusiness’ COVID-19-linked labor recruitment issues as seen in Indonesia? How will pandemic-incurred debts, as observed in Kyrgyzstan, influence future migration patterns and remittance dynamics?

Second, while the direct health impacts had immense consequences for the people affected, the indirect effects of mobility restrictions and economic disruptions were the main causes of the cascading impacts that affected land use and land governance (Fig. 2). These disparate impacts reinforce the importance of the continued need to investigate how manifold connectivities within landscapes, telecouplings between land systems, and the spillover effects of such couplings, influence land-use change and land-use decisions at local, subnational, national, and regional scales (Butler et al. 2022; Meyfroidt et al. 2022).

Third, the fact that the COVID-19 pandemic has had impacts on multiple dimensions requires more in-depth attention to the dynamics of winners and losers of global shocks and how they evolve over time. Will the observed pandemic impacts continue to reflect differential access to resources at different scales, from the intra-urban (Kim and Bostwick 2020; Spotswood et al. 2021) to global (Laborde et al. 2021)? Or will they be redistributed, producing new patterns of access to and exclusion from resources and governance dynamics, as was observed in Argentina and the Amazon for unequal access to digitalization? Will the loss of income produced through international tourism—as seen in Madagascar—or reduced budgets for conservation—as seen in El Chaco and the Amazon—be temporary or permanent? What will be the long-term effects—both positive and negative—of disrupted tourism streams to protected areas, and what are potential lessons learned from the pandemic years that could feed into the development of sustainable tourism? On the other hand, the loss of distant connections can foster new forms of local solidarity and resistance, as observed in the conservation networks in the Amazon. Adding to recent discussions on social-ecological inequalities in the telecoupling literature (Martín-López et al. 2019; Boillat et al. 2020), the COVID-19 pandemic represents a new context to explore how new and old global connections shape social-ecological inequalities and the mechanisms that underpin them.

Fourth, these shifting relations of inequalities also point to the variation and differentiated levels of social-ecological resilience in and between land systems. Here it will be important to understand which attributes associated with resilience have influenced the capacities to cope with and adapt to the shocks resulting from the pandemic, such as shocks to ecosystem services (cf. Pamukcu-Albers et al. 2021; Butler et al. 2022). How does previous exposure to cumulative shocks and multiple hazards (cf., Drakes and Tate 2022) influence the resilience of land systems to the pandemic shocks? How have pandemic shocks interacted with natural hazards and co-occurring stressors to shape landscapes? To what extent can resilience strategies that proved successful during the pandemic be applied to other types of shocks (e.g., natural hazards, climate change) that can be expected in the future? The magnitude and spread of the pandemic shocks offer a unique opportunity to explore aspects related to resilience and to test theoretical concepts through comparative approaches.

Finally, these novel and unanticipated system configurations highlight the need to question whether current indicators (e.g., health, sustainability, economy) are sufficient to characterize system dynamics. For instance, new robust indicators of land system resilience that permit near-real-time measurements of land system dynamics are needed (Wu 2012, 2021). More broadly, to what extent do the experiences of the past many months challenge the relevance of the current Sustainable Development Goals (SDGs)?

The global shocks of the COVID-19 pandemic have affected land systems across the world in similarly direct but differently indirect ways. These systemic shocks offer an unusual opportunity for land scientists to deepen their understanding of the coupled human-natural systems they study. We have started to look at the immediate and longer term effects in our land systems of interest, and we invite our colleagues to consider doing likewise so that we can engage in broader and deeper comparative analyses to increase mutual understanding and identify common vulnerabilities and possible solution pathways for better land stewardship.

## Supplementary Information

Below is the link to the electronic supplementary material.

Supplementary file1 (DOCX 107 kb)
