# Supplementary material for: Global shocks, cascading disruptions, and (re-)connections: viewing the COVID-19 pandemic as concurrent natural experiments to understand land system dynamics
Source: Landsc Ecol. 2023 Mar 2;38(5):1147–61. doi: 10.1007/s10980-023-01604-2 (PMC9977478; doi:10.1007/s10980-023-01604-2)
Supplement: Supplementary file 1 — Supplementary file1 (DOCX 107 kb) [file 10980_2023_1604_MOESM1_ESM.docx]

**Supplementary Material**

***Global shocks, cascading disruptions, and (re-)connections: Viewing the COVID-19 pandemic as concurrent natural experiments to understand land system dynamics***

**S1. VIGNETTES**

**S1.1. Amazon Basin: Peru and Colombia**

Both Colombia and Peru were severely hit by the COVID-19 pandemic, and recorded for some time some of the highest numbers of cases and deaths worldwide (Matheiu *et al.* 2022). The countries also implemented stringent and long-lasting restrictions to contain the pandemic, including lockdowns, international and national mobility restrictions and school closures (Matheiu *et al.* 2022).

This case study focuses on two systems of protected areas: an extensive protected area network in southeastern Peru (Manu National Park (NP), Alto Purus NP, Amarakaeri Communal Reserve, Bahuaja-Sonene NP, Tambopata National Reserve and their buffer areas) and Chiribiquete NP and its greater landscape (including Cahuinarí NP) in southern Colombia, containing some of the largest protected areas in the countries (*e.g.*, Alto Purús NP over 2,510,694 ha, SERNANP 2019, Manú NP: 1,716,296 ha, UNEP-WCMC 201, Chiribiquete NP: 2,782,354 ha, UNESCO 2018).

These areas are recognised for their extraordinary biodiversity (UNEP-WCMC 2011; UNESCO 2018) and are home to local communities who rely mostly on agriculture and trade and several indigenous groups, some of which remain uncontacted (UNEP-WCMC 2011; UNESCO 2018; Parques 2022; Córdoba-Guzmán and Canahuire-Marca 2018). Even though these areas have remained largely intact, anthropogenic threats to conservation are intensifying, especially in their buffers, through the construction of roads, alluvial gold mining, agriculture and cattle expansion as well as drug cultivation and trafficking (FZS 2022). In the Colombian areas, these threats and forest governance have been historically associated with the internal armed conflicts, with conservation areas overlapping areas used and contested by armed groups (Amador-Jimenez *et al.* 2020). Just before the pandemic was declared, the peace accord between the Colombian state and the guerrilla groups encountered difficulties, which forced government park authorities to abandon its offices and checkpoints early in 2020 due to the worsening security situation (FZS 2021b).

The protected area network studied in southeastern Peru expands from the tropical Andes to the Amazon basin. It encompasses conservation areas with various national and international designations, including national parks (under the management authority of SERNANP, the National Service of Natural Areas Protected by the State), communal reserves (co-managed by representatives of indigenous communities), biosphere reserves, and World Heritage Sites. Chiribiquete NP in Colombia is managed by the government authority “Parques Nacionales Naturales de Colombia”. To the South of Chiribiquete lies Cahuinarí NP, which overlaps in its totality the indigenous area “Resguardo Predio Putumayo” (Parques Nacionales Naturales de Colombia 2022), thus requiring all management actions to be approved both by the indigenous and government authorities (Parques Nacionales Naturales de Colombia 2022). International conservation actors such as NGOs provide technical and/or financial support to all of these areas.

**S1.2. Dry Chaco, Argentina**

The “Dry Chaco” case study is based on agroecosystems of the Argentine Dry Chaco ecoregion. The Argentine Dry Chaco is a sedimentary plain that encompasses 490,000 km^2^ in Argentina. It covers 62% of the Gran Chaco biome (786,791 km^2^), which extends north through Paraguay and into southern Bolivia and which is the world’s largest continuous tropical dry forest region. In the Dry Chaco, Seasonal average temperatures range from 28 °C and 16 °C in January and July, respectively. Annual rainfall ranges between 300 and 1000 mm, mostly occurring between November and April (Minetti 1999). Natural woody vegetation is dominated by red quebracho (*Schinopsis lorentzii*), white quebracho (*Aspidosperma quebracho-blanco*), palosanto (*Bulnesia sarmiento*i), and mistol (*Ziziphus mistol*) trees interspersed by shrublands, grasslands, and savannas (Prado 1993).

The Dry Chaco harbors high biodiversity, but it is also a global deforestation hotspot with scarce protected areas, thus many species are currently under threat (Kuemmerle *et al.* 2017). Over the last several decades, deforestation for agriculture and pastureland has expanded rapidly in the Argentine Dry Chaco, and more than 20% (~10 million ha) of its natural areas have already been converted (Vallejos *et al.* 2015). Soybean cultivation is a direct driver of deforestation in parts of the Argentine Chaco, but cattle ranching dominates in areas marginally suited for agriculture (Fehlenberg *et al.* 2017). This pattern is reinforced by regional land-use zoning, which prevents complete forest replacement by crop or pastures in certain areas, but allows for partial clearing, such as the implementation of silvopastoral systems in natural forests (Piquer-Rodríguez *et al.* 2015), which are becoming more widespread across the region. Cattle production is a key economic activity with a long history in the Chaco and it is characterized by a broad diversity of actors, complex management regimes and intensities, and dynamic land tenures (Fernández *et al.* 2020). Additionally, large extensions of forests in the Argentine Dry Chaco are inhabited by traditional small-scale subsistence ranchers (typically called ‘*criollos’*), and by indigenous communities, who are mostly located towards the northern Dry Chaco. *Criollos* raise livestock (bovines, caprines, and ovines) that graze extensively within native vegetation patches surrounding their households (typically called *“puestos”*). Some of these *puestos* have a long family history extending back more than four generations, and have evolved into small communities that rely heavily on a range of social and ecological services provided by the forests (Gasparri 2016). The Dry Chaco is thus very heterogeneous in terms of land-use and stakeholders.

**
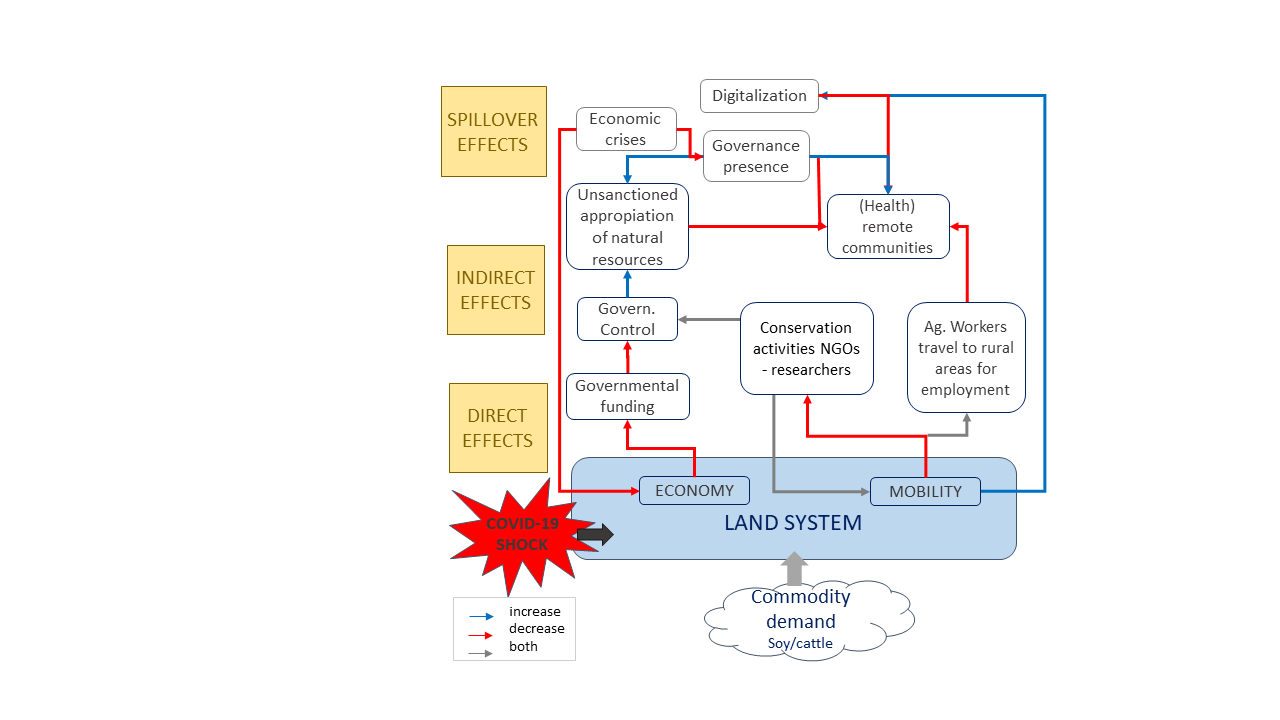
**

Figure S1 provides a schematic representation of expert-based perceived-effects of the COVID-19 shock on the Dry Chaco land system. This kind of specific representation served as initial focus for our discussions prior to the generalized representation of Figure 2 in the main text.

**S1.3. Maroantsetra, Madagascar**

The administrative district of Maroantsetra in Northeastern Madagascar is a major global hotspot for biodiversity (Morelli *et al.* 2019), while at the same time a hub for the production of globally-traded cash crops (e.g. vanilla and clove). Altogether though, it is largely dominated by subsistence agriculture, including shifting cultivation for rice production, which is considered the major direct cause of forest loss and fragmentation (Zaehringer *et al.* 2015). The area supports some of the largest tracts of evergreen rainforest on the island (Vieilledent *et al.* 2018), and hosts some of the largest terrestrial protected areas (PAs) of the country (Goodman *et al.* 2018), such as Makira Natural Park, and Masoala National Park, a global flagship for biodiversity conservation since it was established in 1997 (Kremen *et al.* 1999). The district is home to an estimated 270,000 inhabitants as of 2018 (OCHA 2019), with an overwhelming majority heavily reliant on natural resources for meeting subsistence and income needs (Zaehringer *et al.* 2017). Forest ecosystem services, such as hydrological cycle regulation or erosion control (Llopis *et al.* 2021), are particularly critical to support traditional agricultural activities, such as irrigated rice cultivation, and in many occasions, forests themselves are considered as a reserve of agricultural land to expand subsistence shifting cultivation (Keller 2008). Clove and especially vanilla cultivation and trade are the main income sources for local populations (Andriatsitohaina *et al.* 2020). Vanilla, for which Madagascar is the major world exporter (United Nations 2022), experienced an international price boom until 2018 (Terazono 2017), after which prices dropped significantly (Aust and Hachmann (Canada) Ltd 2020). The specific further implications of this income reduction for local populations remains unclear, but it could be expected that it would lead to a renewed increased reliance on subsistence agricultural activities.

The area has experienced several external shocks in recent years affecting the relation between local populations and the natural resources in the area. A major example is the political crisis of 2009, when former Madagascar President Marc Ravalomanana was ousted from power. This lead to a governance vacuum and thereby to a reduction of monitoring and law enforcement in remote conservation areas, such as the National Parks of Masoala and Marojejy. During the most severe crisis years (2009-2010), these PAs experienced an intensification of unsanctioned exploitation of threatened precious woods for export markets, especially rosewood (*Dalbergia* spp.) and ebony (*Diospyros* spp.) (Randriamalala and Liu 2010; Anonymous 2018). Further, in recent years the area has also experienced several exploitation booms of rare minerals, such as rock crystal, which have had some non-negligible impacts on the environment (Holmes 2007). The area also receives the frequent impact of tropical cyclones making landfall on the east coast, which has caused widespread destruction of lives, houses, and agricultural assets in the past (Brown 2009; Llopis *et al.* 2019), the last being Cyclone Enawo in March 2017 (Le Bellec 2018). However and luckily, despite the area being mildly affected by the impact of cyclones Herold and Eloise in March 2020 and January 2021 respectively, the area have not seen the significant impact of major cyclones during the pandemic years so far (2020-2021) which would have aggravated the already complicated situation. On these already complex dynamics, the COVID-19 pandemic might be having intertwined impacts, to which we refer in the main text of this article.

**S1.4. Besulutu, Indonesia**

Both of the villages in Besulutu, Indonesia studied here are comprised of Tolaki people Indigenous to the region and individuals of predominantly Buginese or Javanese ethnicity that have migrated into Besulutu, particularly since the 1980s. Historically, land systems in Besulutu included extensive wetland complexes managed as permanently inundated swamp forests, seasonally flooded marshes, and riparian zones for vegetable production, fishing, swamp timber harvest, sagu palm production, and other domestic and medicinal needs (Tarimana 1989; de Jong 2017). These predominantly dry season production systems complemented swidden agricultural practices in hillside agro-forests during rainy months of the year (wherein land was generally used to produce a rice and corn crop for two to three years before fallowing the land and planting tree and fruit crops to encourage its long-term regrowth and serve various long-term productive and reproductive purposes, *e.g.*, access to timber, fuel wood, and seasonal fruit harvests (Tarimana 1989). Landscapes as of 2022 are instead dominated by corporate oil palm production, intensive smallholder tree crop production, industrial mining, and state production forests with relatively few remaining wetlands and agroforests on the landscape (Dean 2021).

Over a century of colonial, state-led and neoliberal developments have been critical in effecting these transformations in Besulutu’s land systems, as have the resistances and strategic forms of re- and de-agraraianization individuals living in the area have engaged in. Among other things, for instance, the area was the site of several state resettlement schemes from the late 1960s through the late 1970s, including a failed local transmigration scheme in 1969 that attempted to develop the flooded swamp lands for rice production as part of a broader push to sedentarize mobile swidden livelihoods. Settlement in Besulutu and the sedentarization of swidden livelihoods were also encouraged through block grants to support smallholder cacao production and other tree crops through the late 1990s and early 2000s (Kelley 2018). Changes in land use, cover, and control over the past two decades have also been defined by the legacy of state land claims in the area (which are strongest to the northeast of the sub-district where they are formalized as part of state production forest) and by the growth in industrial concessions for both oil palm and mining in the province since 2009, and in the study area since roughly 2011 in the form of corporate palm (to the south) and corporate nickel (to the north). As elsewhere in Sulawesi and Indonesia (*e.g.*, Voight *et al.* 2021), the expansion of such claims has engendered significant tree cover loss, particularly due to oil palm expansion into many remaining smallholder-held agro-forests between 2011-2017 (Dean 2021).

Many households in the area nonetheless continue to engage in land management on remaining fields and farms, with most families retaining access to at least one small plot of land for food and/or commodity production, particularly corn, cacao, peppercorn, and other vegetable and tree crops. While individuals in the area have long been dependent on seasonal work outside the sub-district, the dependency of many households on extra-local labor migration has increased and individuals increasingly travel further and for longer periods of time. The first international labor migration (to Malaysia) also appears to have occurred in the late 1990s. Prior to the effects of mobility restrictions beginning in March 2020 in this area, most people (particularly men between the ages of 15-35) were migrating to sites of work in nearby peri-urban or urban areas or to other primary industries in Indonesia (*e.g.*, logging, mining, or plantation economies in Ambon, Papua, and Kalimantan). The expansion of localized wage labor opportunities on the plantation and nickel mine affect such rates and patterns of migration and change local landscapes, albeit in ways that remain weakly understood both locally and in village-concession landscapes elsewhere in Indonesia (Kelley *et al.* 2022), particularly as these patterns have changed considerably throughout the course of the oil palm and mining concession’s first 10 years of operation.

**S1.5. Naryn, Kyrgyzstan**

Kyrgyzstan is a small lower and middle income country in Central Asia that emerged from the dissolution of the Soviet Union three decades ago. Its geographic area is a bit less than 200,000 sq km, of which more than 90% is mountainous and inland waters cover 4%. Of the 191,800 sq km of land area, 7% is forested, croplands cover just 7%, and 47% in pastures. The balance of the remaining lands are primarily barren areas and permanent snow and ice. There are few large cities and most people live in rural areas. The rugged terrain restricts large scale agricultural production to few lowland areas. The basis of the rural economy is a form of agropastoralism that features the seasonal movement (transhumance) of livestock (*e.g.*, sheep, cattle, horses, yaks, *etc.*) to more distant, higher elevation pastures coupled with cultivation of forage crops for winter feed. While the economy of Kyrgyzstan is notable for its heavy reliance on international remittances, domestic remittances are also significant (Wang *et al.* 2021).

The impact of COVID-19 on international remittances to Kyrgyzstan was not as severe as predicted in late 2020 (World Bank 2020). The two sharpest annual decreases in remittances to Kyrgyzstan occurred between 2014 and 2015 (-25%) in the wake of economic sanctions on Russia after annexation of Crimea and between 2008 and 2009 (-20%) following the onset of the global recession (World Bank-KNOMAD 2022). Thus, the personal telecoupling through remittances between international migrants–working primarily in Russia–and their families back in rural Kyrgyzstan exhibited resilience despite economic slowdowns. There is speculation that some of the remitted funds that were previously hand-carried into the country were transmitted instead through the international banking systems due to mobility restrictions and lockdowns (Ratha *et al.* 2020), which could account for the observed modest increase in remittances to Kyrgyzstan in 2020.

**S1.6. Trento, Italy**

The analysis of the urban system of Trento focused on the availability of green spaces as a critical feature that affected the physical and mental wellbeing of urban population during the pandemic. An increase in park visitation was a common pattern across many cities around the world, indicating a growing importance of contact with nature for both recreation and restoration (Volenec *et al*. 2021). However, to ensure physical distancing and reduce the risk of disease transmission, many national and local administrations decided, in certain periods, to close or restrict access to public green areas, for example by limiting the distance that people were allowed to travel from home (Ugolini *et al.* 2020). These measures put a disproportionate burden on the vulnerable groups that rely more on the benefits provided by public green spaces, and their effects were highly controversial (Slater *et al.* 2020; Pouso *et al.* 2021). To support the formulation of evidence-based policies, the city of Trento was used as a case study in Geneletti *et al.* (2022) to explore, through simulations, the potential effects of alternative strategies aimed at reducing the spread of COVID-19.

Trento is a middle-size city of around 120,000 located in north-eastern Italy. The main urban settlement, which hosts about 70% of the population, lies in the valley floor at around 200 m asl, surrounded by hills and mountains that rapidly reach an elevation of more than 2,000 m. The main transport infrastructure, as well as most industrial and commercial areas, are also located in the valley floor. Vineyards and apple orchards occupy most of the sunny hill-sides, while the majority of the administrative area is covered by forests. The distribution and characteristics of public green spaces reflect traditional planning regulations aimed at providing all residential districts with small local parks. Larger green spaces, predominantly located in peri-urban areas, are partly the result of protection regimes aimed at safeguarding a combination of biodiversity and cultural values, partly the results of recent efforts of the administration to increase the provision of green areas in some densely-populated neighborhoods (Cortinovis *et al.* 2018). The analysis considered the 98 urban parks of the city, defined as public green areas larger than 300 m2 designed and managed for citizens’ access and recreational use. Overall, they cover 93.6 ha, equivalent to 3.1% of the urban area, corresponding to about 8 m2 per inhabitant. Of them, 61 are local parks smaller than 0.5 ha, while 10 are large parks bigger than 2 ha, including 3 city-level parks bigger than 10 ha. The size usually corresponds to the availability of facilities, with smaller parks normally equipped with playground and benches, and larger parks offering opportunities for a wider range of activities, including sports, and a more direct contact with nature.

The simulated policies are a combination of three variables: i) type of green areas that are made accessible to the public; ii) maximum distance that people are allowed to travel from home to reach the green space; and iii) access restrictions targeting specific sectors of the population. In terms of the type of green areas, we considered the options of using only the existing urban parks (status quo), or increasing the availability of public green spaces by opening schoolyards to public use as an emergency response to the pandemic. In terms of distance, we considered four thresholds based on common international standards for green space planning (Stessens *et al.* 2017), as well as on the restrictions enforced by some national and regional governments during the different phases of the pandemic: 200 m, 300 m, 400 m, and 500 m. Finally, in terms of restrictions for specific population groups, we simulated the case in which everyone is allowed to access the closest green area, and the case in which only people without a private garden have access to public green spaces. Whilst not based on existing regulations, the latter case would be justified from an equity perspective, since people who can access a private garden do not have the same need of visiting a public park as people who live in the denser neighborhoods of the city, hence it might help to identify priorities for intervention.

By combining all the possible states of the three variables, we generated 16 policy scenarios, whose effects were modelled through GIS algorithms and analyzed using two indicators: the number of people with access to a green area, and the share of people with access to an uncrowded green area.. The complete results, reported in Geneletti *et al.* (2022), support the selection of effective emergency interventions to cope with the pandemic (such as the opening of schoolyards), but they also reveal hotspots of needs that require medium-to-long term planning strategies to improve the current distribution of green spaces in the city. The knowledge gained through this and similar experiences can be used to plan and manage urban green spaces in ways that are more resilient to unexpected conditions.

**S2. SUPPLEMENTAL REFERENCES**

Amador-Jiménez M, Millner N, Palmer C, Pennington RT, Sileci L (2020). The unintended impact of Colombia’s COVID-19 lockdown on forest fires. Environ Res Econ 76(4):1081-1105

Andriatsitohaina RNN, Celio E, Llopis JC, Rabemananjara ZH, Ramamonjisoa BS, Grêt-Regamey A (2020) Participatory Bayesian network modeling to understand driving factors of land-use change decisions: insights from two case studies in northeast Madagascar. J Land Use Sci 15(1):69-90

Anonymous (2018) Rosewood democracy in the political forests of Madagascar. Polit Geogr 62:170-183

Aust and Hachmann (Canada) Ltd (2020). Vanilla market update – November 2020. Retrieved 25 November 2020 from<http://www.austhachcanada.com/november-2020/>

Brown ML (2009) Madagascar’s Cyclone Vulnerability and the Global Vanilla Economy. The Political Economy of Hazards and Disasters. EC Jones, AD Murphy, eds. AltaMira Press: Plymouth, UK. pp 241-264

Córdoba-Guzmán D, Canahuire-Marca N.(2018) Estudio socioeconomico de la asociacion de productores de Callanga. 147 pp.

Cortinovis C, Zulian G, Geneletti D (2018). Assessing nature-based recreation to support urban green infrastructure planning in Trento (Italy). Land 7(4):112 <https://doi.org/10.3390/land7040112>

Dean J (2021) Effects of state enclosures and industrial concessions on land cover change in
Indonesia. Masters' Thesis. University of Hawai'i at Manoa, Department of Geography and
Environment.

de Jong CGF (2017). Nieuwe hoofden, Nieuwe goden. Geschiendenis van de Tolaki en de Tomoronene, twee volkeren in Zuidoost-Celebes (Indonesie), tot ca. 1950. ISBN 978-3-8443-8756-8.

Fehlenberg V, Baumann M, Gasparri NI, Piquer-Rodriguez M, Gavier-Pizarro G, Kuemmerle T (2017) The role of soybean production as an underlying driver of deforestation in the South American Chaco. Global Environl Change 45:24–34 <https://doi.org/10.1016/j.gloenvcha.2017.05.001>

Fernández P D, Kuemmerle T, Baumann M, Grau HR, Nasca JA, Radrizzani A, Gasparri NI (2020) Understanding the distribution of cattle production systems in the South American Chaco. J Land Use Sci 15(1):52-68

FZS (Frankfurt Zoological Society) (2021b) Annual report 2020. 76 pp. Retrieved 27.03.2022 from <https://fzs.org/en/news/annual-report-2020/>

FZS (Frankfurt Zoological Society) (2022) Frankfurt Zoological Society - Projects. <https://fzs.org/en/projects/>

Gasparri NI 2016. The transformation of land-use competition in the Argentinean Dry Chaco between 1975 and 2015. In Land Use Competition (Niewöhner et al. eds.). Springer, Berlin, DE. pp 59-73

Geneletti D, Cortinovis C, Zardo L (2022) Simulating crowding of urban green areas to manage access during lockdowns. Landscape Urban Plan 219:104319 <https://doi.org/10.1016/j.landurbplan.2021.104319>

Goodman SM, Raherilalao MJ, Wohlhauser S (2018) Les Aires Protégées Terrestres de Madagascar : Leur Histoire, Description et Biote / The Terrestrial Protected Areas of Madagascar: Their History, Description and Biota. Antananarivo, Madagascar, Association Vahatra, p 1716

Holmes C (2007) Linking livelihoods, land stewardship, and resource conservation in the Antongil Bay landscape, Madagascar. Protected areas and human livelihoods (K. Redford and E. Fearn, eds.). Wildlife Conservation Society, New York (Working Paper 32), 6-16

Keller E (2008). The banana plant and the moon: Conservation and the Malagasy ethos of life in Masoala, Madagascar. Am Ethnol 35(4):650-664

Kelley LC (2018) The politics of uneven smallholder cacao expansion: A critical physical geography of agricultural transformation in Southeast Sulawesi, Indonesia. Geoforum 97:22-34

Kelley LC, Shattuck A, Thomas K (2022) Cumulative socio-natural displacements: Reconceptualizing climate displacements in a world already on the move. Ann Am Assoc Geogr 112(3):664-673 <https://doi.org/10.1080/24694452.2021.1960144>

Kremen C, Razafimahatratra V, Guillery RP, Rakotomalala J, Weiss A, Ratsisompatrarivo J-S (1999) Designing the Masoala National Park in Madagascar based on biological and socioeconomic data. Conserv Biol 13(5):1055-1068

Kuemmerle T. Altrichter M, Baldi G, Cabido M, Camino M, Cuellar E, ... Zak M (2017). Forest conservation: Remember Gran Chaco. Science 355(6324):465-465

Le Bellec A (2018) North-Eastern Madagascar and Cyclone Enawo. A discussion of the concept of resilience. The State of Environmental Migration 2018: A review of 2017. C. Zickgraf, E. Hut and F. Gemenne. Liège, Presses Universitaires de Liège:11-34.

Llopis JC, Chastonay JF, Birrer FC, Bär R, Andriatsitohaina RNN, Messerli P, Heinimann A, Zaehringer JG (2021) Year-to-year ecosystem services supply in conservation contexts in north-eastern Madagascar: Trade-offs between global demands and local needs. Ecosys Services 48:101249

Llopis JC, Harimalala PC, Bär R, Heinimann A, Rabemananjara ZH, Zaehringer JG (2019) Effects of protected area establishment and cash crop price dynamics on land use transitions 1990–2017 in north-eastern Madagascar. J Land Use Sci 14(1):52-80

Mathieu E, Ritchie H, Rodés-Guirao L, Appel C, Giattino C, Hasell J, Macdonald D, Dattani S, Beltekian D, Ortiz-Ospina E, Roser M (2020) "Coronavirus Pandemic (COVID-19)". Published online at OurWorldInData.org. Retrieved from: <https://ourworldindata.org/coronavirus> 05JAN2023.

Minetti JL (1999) Atlas Climático del Noroeste Argentino. Laboratorio Climatológico Sudamericano, Fundación Zon Caldenius, Tucuman, Argentina

Morelli TL, Smith AB, Mancini AN, Balko EA, Borgerson C, Dolch R, ... Baden AL (2020). The fate of Madagascar’s rainforest habitat. Nature Clim Change 10(1):89-96

OCHA (2019). Population, administrative level 0 - 4 boundaries and road data, Madagascar. United Nations Office for the Coordination of Humanitarian Affairs (OCHA). <https://data.humdata.org>

Parques Nacionales Naturales de Colombia (2022) Parques Nacionales Naturales de Colombia – Parques Nacionales. Retrieved 26.03.2022 from<https://www.parquesnacionales.gov.co/portal/es/parques-nacionales>

Piquer-Rodríguez M, Torella S, Gavier-Pizarro G, Volante J, Somma D, Ginzburg R, Kuemmerle T (2015) Effects of past and future land conversions on forest connectivity in the Argentine Chaco. Landscape Ecol 30(5):817-833

Pouso S, Borja Á, Fleming LE, Gómez-Baggethun E, White MP, Uyarra MC (2021) Contact with blue-green spaces during the COVID-19 pandemic lockdown beneficial for mental health. Sci Total Env 756:143984 <https://doi.org/10.1016/j.scitotenv.2020.143984>

Prado D (1993) What is the Gran Chaco vegetation in South America? I. A review. Contribution to the study of flora and vegetation of the Chaco. V. Candollea 48:27

Randriamalala H, Liu Z (2010) Rosewood of Madagascar: Between democracy and conservation. Madagascar Cons Dev 5(1):11-22

Ratha D, De D, Kim EJ, Seshan G, Yameogo ND, Plaza S (2020) COVID-19 crisis through a migration lens (Migration and development brief 32). World Bank Group. <http://documents.worldbank.org/curated/en/989721587512418006/COVID-19-Crisis-Through-a-Migration-Lens>.

SERNANP (Servicio Nacional de Areas Naturales Protegidas por el Estado) (2019) Plan Maestro Parque Nacional Alto Purús 2019-2023*.* [Retrieved from https://old.sernanp.gob.pe/sernanp/archivos/baselegal/Resoluciones_Presidenciales/2019/RP%20N%20087-2019-COMPLETO.pdf](https://old.sernanp.gob.pe/sernanp/archivos/baselegal/Resoluciones_Presidenciales/2019/RP%20N%20087-2019-COMPLETO.pdf)

Slater SJ, Christiana RW, Gustat J (2020) Recommendations for keeping parks and green space accessible for mental and physical health during COVID-19 and other pandemics. Prev Chron Dis 17:200204 <https://doi.org/10.5888/pcd17.200204>

Stessens P, Khan AZ, Huysmans M, Canters F (2017) Analysing urban green space accessibility and quality: A GIS-based model as spatial decision support for urban ecosystem services in Brussels. Ecosys Serv 28:328-340 <https://doi.org/10.1016/j.ecoser.2017.10.016>

Tarimana A (1989) Kebudayaan Tolaki (Tolaki Culture), Seri Etnografi Indonesia No. 3. Balai Pustaka, Jakarta

Terazono E (2017) Vanilla price reaches record high after Madagascar cyclone. Financial Times. Retrieved 20.12.2021 from<https://www.ft.com/content/e0e2fc16-28db-11e7-bc4b-5528796fe35c>

Ugolini F, Massetti L, Calaza-Martínez P, Cariñanos P, Dobbs C, Ostoić SK, ..., Sanesi G (2020) Effects of the COVID-19 pandemic on the use and perceptions of urban green space: An international exploratory study. Urban For Urban Green 56:126888. <https://doi.org/10.1016/j.ufug.2020.126888>

UNEP-WCMC (2011) *World Heritage Site Datasheet – Manu National Park.* [Retrieved 27.03.2022 from http://world-heritage-datasheets.unep-wcmc.org/datasheet/output/site/manu-national-park/](http://world-heritage-datasheets.unep-wcmc.org/datasheet/output/site/manu-national-park/)

UNESCO (2018) *World Heritage Centre – World Heritage List - Chiribiquete National Park – The Maloca of the Jaguar.* Retrieved 27.03.2022 from<https://whc.unesco.org/en/list/1174/>

United Nations (2022) *UN comtrade*. Retrieved 20.01.2022, from<http://comtrade.un.org/>

Vallejos M, Volante JN, Mosciaro MJ, Vale LM, Bustamante ML, Paruelo JM (2015) Transformation dynamics of the natural cover in the Dry Chaco ecoregion: A plot level geo-database from 1976 to 2012. J Arid Env 123:3–11 <https://doi.org/10.1016/j.jaridenv.2014.11.009>

Vieilledent G, Grinand C, Rakotomalala FA, Ranaivosoa R, Rakotoarijaona J-R, Allnutt TF, Achard F (2018) Combining global tree cover loss data with historical national forest cover maps to look at six decades of deforestation and forest fragmentation in Madagascar. Biol Conserv 222:189-197

Voigt M, Supriatna J, Deere NJ, Kastanya A, Mitchell SL, Rosa IM, Santika T, Siregar R, Tasirin JS, Widyanto A, Winarni NL (2021). Emerging threats from deforestation and forest fragmentation in the Wallacea centre of endemism. Environ Research Lett 16(9):094048

Volenec ZM, Abraham JO, Becker AD, Dobson AP (2021) Public parks and the pandemic: How park usage has been affected by COVID-19 policies. PLoS ONE 16(5):e0251799 <https://doi.org/10.1371/journal.pone.0251799>

Wang D, Hagedorn A, Chi G (2021) Remittances and household spending strategies: evidence from the Life in Kyrgyzstan Study, 2011–2013. J Ethnic Migr Stud 47(13):3015-3036

World Bank (2020) World Bank Predicts sharpest decline of remittances in recent history. <https://www.worldbank.org/en/news/press-release/2020/04/22/world-bank-predicts-sharpest-decline-of-remittances-in-recent-history>

World Bank-KNOMAD (2022) Inward Remittance Flows. Online: <https://www.knomad.org/sites/default/files/2022-12/inward_remittance_flows_as_of_dec._2_2022_0.xlsx>

Zaehringer J, Eckert S, Messerli P (2015) Revealing regional deforestation dynamics in North-Eastern Madagascar—Insights from multi-temporal land cover change analysis. Land 4(2):454 <https://doi.org/10.3390/land4020454>

Zaehringer JG, Schwilch G, Andriamihaja OR, Ramamonjisoa D, Messerli P (2017) Remote sensing combined with social-ecological data: The importance of diverse land uses for ecosystem service provision in north-eastern Madagascar. Ecosystem Serv 25:140-152
